# Ge_14_Br_8_(P^*n*^Pr_3_)_4_: A Material for Laser-Induced Printing of Elemental Germanium

**DOI:** 10.1021/acs.inorgchem.6c00575

**Published:** 2026-05-06

**Authors:** Enes Ünver, William Roberts, Martin Eberle, Kai Braun, Marcus Scheele, Andreas Schnepf

**Affiliations:** 1 Institute of Inorganic Chemistry, 9188 Universität Tübingen, Auf der Morgenstelle 18, Tübingen D-72076, Germany; 2 Institute of Physical and Theoretical Chemistry, 9188 Universität Tübingen, Auf der Morgenstelle 18, Tübingen D-72076, Germany

## Abstract

The continued miniaturization of microelectronic systems requires new strategies for fabricating microelectronic materials beyond conventional lithography and metal evaporation. Metalloid nanoclusters offer promising alternatives for printing and patterning conductive and semiconductive elements. Here, we report on the synthesis, structural characterization, and first application of the soluble metalloid germanium cluster Ge_14_Br_8_(P^*n*^Pr_3_)_4_, obtained via controlled disproportionation of a metastable GeBr solution. Substitution of the phosphine ligands markedly enhances the solubility in organic solvents, enabling comprehensive spectroscopic characterization via nuclear magnetic resonance (NMR), ultraviolet-visible (UV–vis), and mass spectrometry. Furthermore, the cluster demonstrates a high Ge content of 45 wt %, rendering it a possible precursor for laser-induced writing of elemental germanium under inert conditions. To demonstrate its potential, we fabricate deliberately designed patterns of Ge microstructures and assess their semiconducting behavior. This work establishes the first example of direct laser writing of germanium from a soluble metalloid cluster precursor, opening new pathways for additive microfabrication in semiconductor technology.

## Graphical abstract

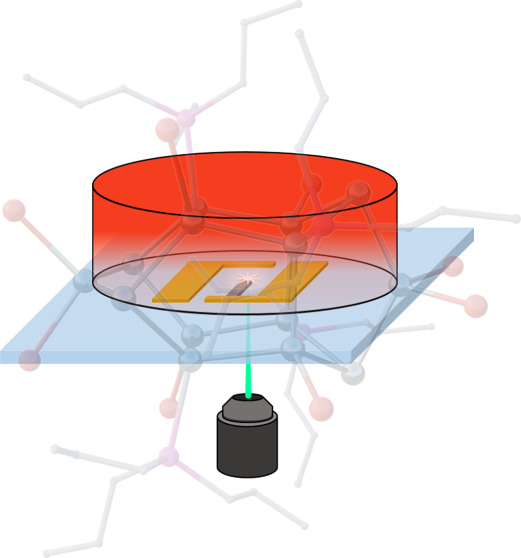

## 1 Introduction

Electronic systems are based on the manipulation of electric currents by conductors and semiconductors. The logical switching of current via electronic components, such as transistors, capacitors, diodes, and resistors, is based on the physical properties of different metals, semimetals, and alloys. Transistors, which are made up of semiconductor components, are the most fundamental building blocks for logical switches in the computer industry. Advances in this field could lead to major breakthroughs in the semiconductor industry.

Unlike the common method of mask lithography and metal evaporation, microelectronics can also be realized by laser material processing, i.e., printing from colloidal dispersions or metal–organic precursor solutions.^1^ Various metals, such as gold, silver, copper, platinum, and aluminum, could be printed through sintering.^2^ In particular, solutions of the metalloid gold cluster Au_32_(P^*n*^Bu_3_)_12_Cl_8_ (**1**) are suitable as a gold ink and demonstrate the potential of these types of compounds.^3^ In the case of the semiconducting element germanium, no suitable precursor molecule was found yet.

In contrast to metal nanoparticles, **1** is an atomically precise metalloid cluster. The arrangement of the metal atoms within such a metalloid cluster compound provides insight into the process of the dissolution and formation of the respective bulk metal. Therefore, the arrangement often represents a cut-out of the metal’s bulk structure. The similarity to the bulk phase might be traced back to the fact that metalloid clusters exhibit naked metal atoms in oxidation state zero and have more metal–metal than metal–ligand contacts.^4,5^ The average oxidation state of the metal atoms within metalloid clusters approaches zero with increasing diameter.^6^

Germanium clusters are typically synthesized through the reductive coupling of organogermanium halides. The common oxidation states of germanium halides are +IV and +II, and the most widely applied compound is GeCl_2_·dioxane, which can be produced under inert conditions and used as a starting reagent for many organogermanium compounds.^7^

Possible synthesis pathways to metalloid germanium clusters are the reductive elimination of ligands and/or the reductive coupling of useful precursors with reducing agents like alkali metals.^8^ However, the most successful synthesis strategy is the disproportionation reaction of subvalent germanium compounds. Therefore, the addition of bulky ligands can stabilize the intermediary formed metalloid clusters during the disproportionation. Since this kinetic stabilization is only effective at low temperatures, GeCl_2_·dioxane is not a useful precursor as its disproporationation requires 150 °C.^9,10^ Therefore, another synthetic approach is needed.

Germanium halides in the oxidation state +I can be synthesized at high temperatures and low pressure in the form of gaseous GeX (X = Cl and Br). Applying the preparative co-condensation technique, metastable solutions of GeX can be obtained in high yields. Therefore, gaseous GeX is co-condensed with a donor and a solvent on a cold metal surface at −196 °C to form a solid matrix. Heating the matrix to −78 °C gives after melting a metastable, donor-stabilized GeX solution, which can be stored at −78 °C, offering a unique reagent for germanium cluster chemistry.^11^ These metastable solutions disproportionate at mild temperatures, finally resulting in the formation of elemental germanium and halides of higher oxidation states such as GeX_2_ or GeX_4_. Upon heating to room temperature, germanium-rich intermediates are formed on the way to elemental germanium. These intermediary formed clusters can be trapped by kinetic stabilization. This kinetic stabilization can be either achieved solely through the choice of the right donor or by substituting the halide ligands with bulky ligands.^12^

In 2015, our group published the metalloid cluster Ge_14_Br_8_(PEt_3_)_4_ (**2**). **2** is synthesized by heating a matrix of GeBr, toluene, and the donor PEt_3_ to room temperature. After removing excess PEt_3_ by washing with pentane and extraction with tetrahydrofuran, red, octahedral shaped crystals of **2** were obtained.

The synthesis and characterization of **2** were breakthroughs in germanium cluster chemistry since it is the first metalloid germanium cluster stabilized by halide and phosphine ligands only. Therefore, **2** is a direct intermediate in the disproportionation cascade of phosphine-stabilized GeBr. It is also the first subhalide germanium cluster with an average oxidation state of the germanium atoms lower than one.^13^ Unfortunately, subsequent investigations of **2** were hindered by the fact that crystals of **2** are insoluble in common solvents. Consequently, follow-up reactions and applications could not be investigated to date.

We reasoned that introducing a donor with larger alkyl chains might increase the solubility and open the door for future investigations. In this work, we present the cluster Ge_14_Br_8_(P^*n*^Pr_3_)_4_ (**3**) with greatly improved solubility and first applications.

## 2 Results and Discussion

Gaseous GeBr is synthesized in a graphite reactor at 1600 °C by the reaction of elemental germanium and HBr at approximately 10^–2^ mbar. The thus obtained gaseous GeBr is co-condensated with an excess of P^*n*^Pr_3_ and THF at −196 °C in a homemade co-condensation apparatus to give a solid matrix.^11^ After the matrix was heated to −78 °C, it melts and a bright red solution is obtained. Heating this solution to room temperature, the disproportionation reaction leads to a color change to give a dark red solution and no precipitate is observed (Figure S1). Hence, in contrast to the orange suspension from which **2** can be crystallized, in the case of P^*n*^Pr_3_ as the donor component, the solution can be heated to room temperature without the formation of any solid precipitate, already indicating a better solubility of the cluster compounds in solution. After heating to room temperature, the solvent was removed, and the dark red residue is washed with pentane. Extraction with acetonitrile and storing of the dark red extract at 6 °C leads to red rod-shaped crystals (Figure S3). X-ray crystal structure analysis reveals that the metalloid cluster Ge_14_Br_8_(P^*n*^Pr_3_)_4_ (**3**) has formed, representing like **2** an intermediate in the disproportionation cascades, and this time of THF/P^*n*^Pr_3_ stabilized GeBr.**3** crystallizes in the orthorhombic crystal system in the space group *P*2_1_2_1_2_1_. Acetonitrile molecules are also present within the crystal, highlighting the influence of the extraction solvent on the crystallization (Figure 1).

**Figure 1:**
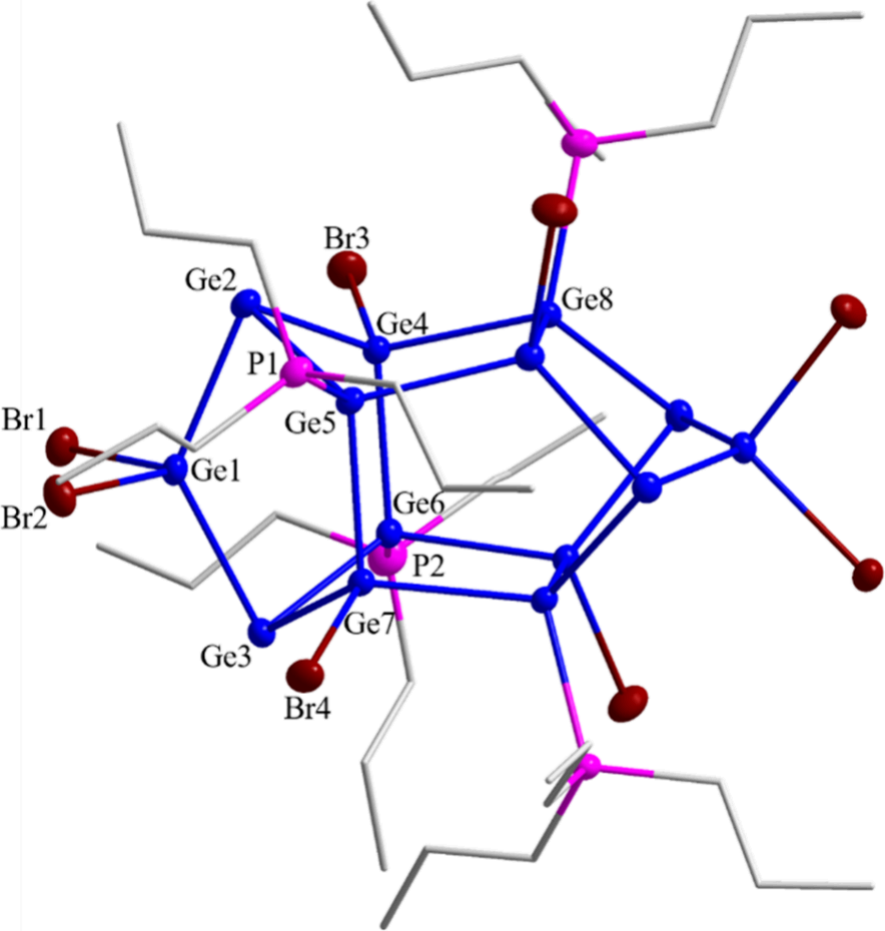
Molecular structure of Ge_14_Br_8_(P^*n*^Pr_3_)_4_ (**3**) without hydrogen atoms. The alkyl chains are shown via a stick presentation. All other atoms are shown as thermal ellipsoids with 50% probability. Selected bond lengths (pm) and angles [°]: Ge1–Ge2: 248.8(1), Ge2–Ge4: 248.45(10), Ge2–Ge5: 251.41(10), Ge4–Ge6: 251.47(9), Ge4–Ge8: 247.33(9), Ge1–Br1: 241.75(11), Ge4–Br3: 238.35(10), Ge5–P1: 235.09(19), Ge2–Ge1–Ge3: 129.50(4), Ge2–Ge5–Ge7: 114.33(4), Ge1–Ge3–Ge6: 85.52(3), Ge1–Ge3–Ge7: 84.20(3), Ge6–Ge3–Ge7: 82.38(3), P1–Ge5–Ge2: 110.01(5), P1–Ge5–Ge7: 111.79­(5), Br1–Ge1–Br2: 100.81(4), Ge3–Ge7–Br4: 108.14(4), and Br1–Ge1–Ge2: 106.79(4).

Looking at the structure of **3**, it is obvious that it is isostructural to **2**. Hence, the structure of **3** can be described as a dimer of two norbornadiene-like Ge_7_-subunits bound together by Ge–Ge single bonds of around 248 pm (Figure 2). Thus, **3** forms an empty polyhedron constructed solely out of shared Ge_5_ rings, which is a structural motif of the clathrate II structure of solid germanium (cF136).^14^

**Figure 2:**
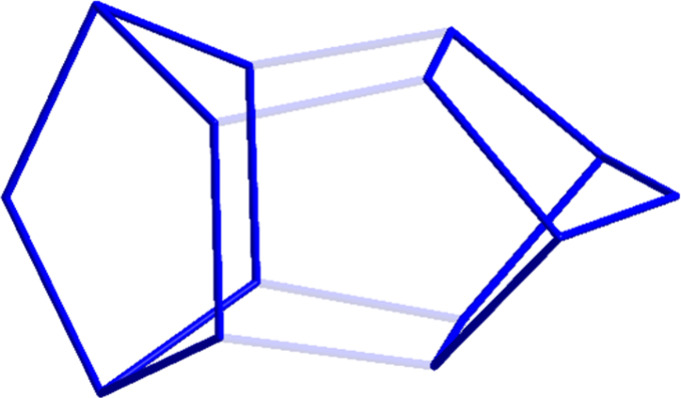
Norbonadiene subunits of the germanium cage of **3**.

Regarding the interatomic distance within **3**, the Ge–Ge bonds vary in the range from 247.3 to 251.5 pm and are thus comparable to **2** (248.3–250.3 pm). The average Ge–Ge distance is 250 pm, slightly longer than Ge–Ge distances in α-germanium (245 pm). Within **3**, germanium atoms with oxidation states of +II, +I, and −I are present, leading to an average oxidation state of the germanium atoms of 0.57. The average Ge–Br distances within **3** are 241.6 pm within the GeBr_2_ units and 238.8 pm within the GeBr units. These Ge–Br distances are comparable to those of **2**. Ge–P bond distances are 239.4 pm comparable to known Ge–P single bonds but slightly shorter than those found within phosphine-stabilized GeX_2_ (X = Cl and Br) compounds.^15,16^

Crystals of **3** are well soluble in tetrahydrofuran, highlighting the influence of the bound phosphine. This allows the characterization of compound **3** via mass spectrometry and UV–vis and NMR spectroscopy. In the electrospray ionization mass spectrum of the THF solution of **3** (Figure 3 and Figures S4 and S5), we observe in the high mass cationic region the most intense main signal of the protonated cluster [HGe_14_Br_8_(P^*n*^Pr_3_)_4_]^+^. Additionally, we find the cluster [Ge_14_Br_7_(P^*n*^Pr_3_)_4_]^+^, which is obtained by the elimination of one Br^–^ substituent. In both cases, the calculated and simulated isotopic pattern fit perfectly, and the distance of 1 amu between the different isotopologues shows a charge of +1 for both clusters. In the case of [HGe_14_Br_8_(P^*n*^Pr_3_)_4_]^+^, the signal of [(THF)­Ge_14_Br_7_(P^*n*^Pr_3_)_4_]^+^ is superimposed, fitting perfectly to the simulated isotopic pattern. The fact that the main signal in the higher mass region is the protonated compound [HGe_14_Br_8_(P^*n*^Pr_3_)_4_]^+^ indicates that **3** is a good Bro̷nsted base, which is checked by ongoing investigations. The UV–vis spectrum of a THF solution of **3** (Figure S6) shows no discrete absorption bands and only an absorption onset at 650 nm. A similar absorption behavior is known from halide-terminated germanium nanoparticles.^17^

**Figure 3:**
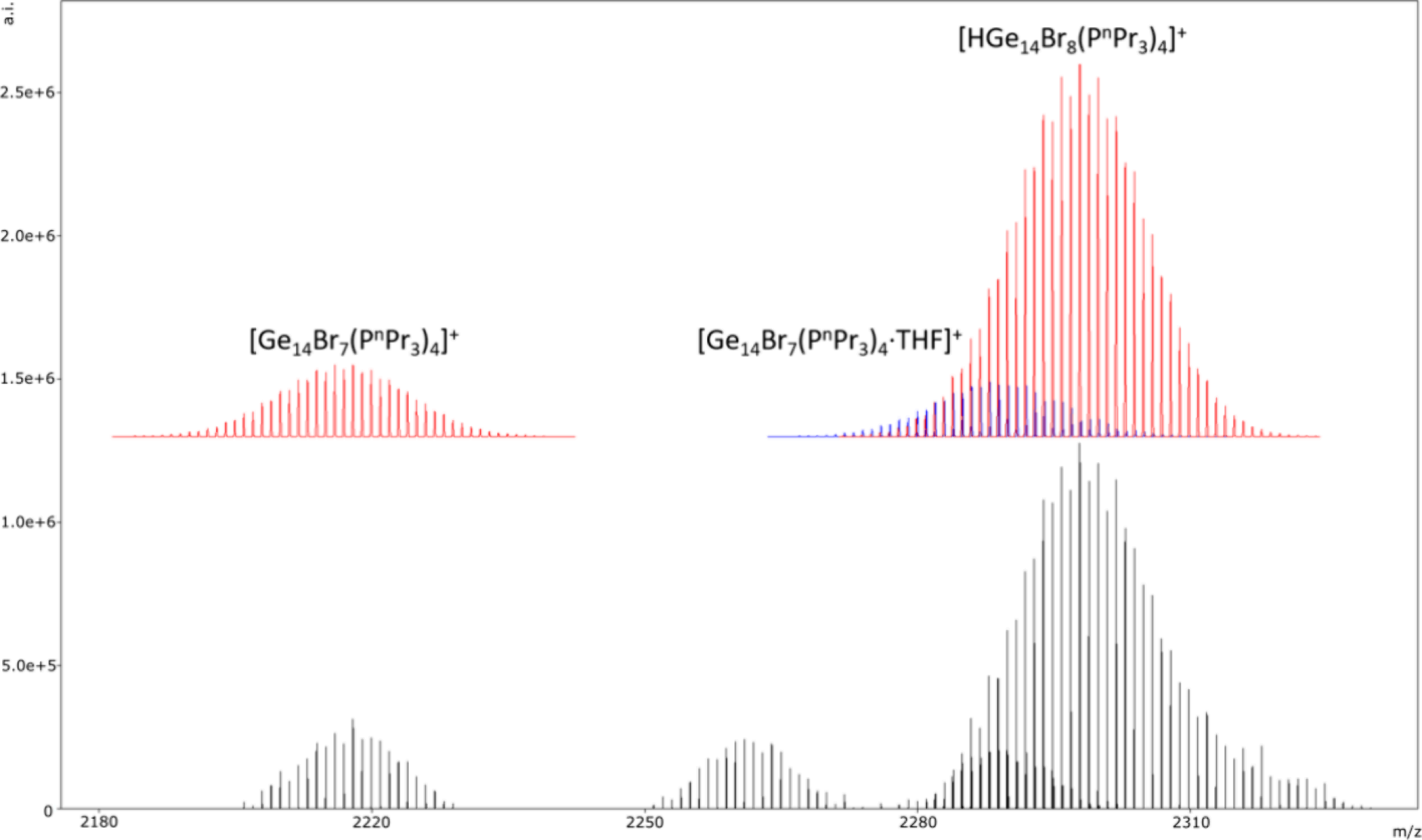
Bottom: High mass region of HR-ESI-APCI-APPI-MS of **3**. Top: Calculated isotopic pattern (red and blue) of the cationic clusters [HGe_14_Br_8_(P^*n*^Pr_3_)_4_]^+^, [Ge_14_Br_7_(P^*n*^Pr_3_)_4_]^+^ (red), and [Ge_14_Br_7_(P^*n*^Pr_3_)_4_·THF]^+^. (blue).

Within the ^31^P NMR spectrum of **3**, only one singlet at 14.1 ppm is observed, as expected since the bound phosphine ligands are symmetry-equivalent. Due to the good solubility of **3** and other intermediates on the way to **3**, variable temperature NMR (VT-NMR) investigations of the metastable GeBr solution (Figure 2) allow first insights into the disproportionation cascade and the formation of **3**. Beginning at −78 °C, many signals are detected, showing that the broad spectrum of the primary formed different compounds in the metastable solution after the melting of the matrix. The signal of **3** thereby appears at about −40 °C and intensifies while heating to room temperature, showing that **3** is one of the major metastable products, formed on heating the solution to room temperature. The other compounds, correlated to the signals at 0 and 5 ppm, could not be identified yet. Nevertheless, the VT-NMR investigations show the intermediary formation of a variety of yet unknown species, which at room temperature lead to a less complex mixture from which **3** can be obtained in crystalline form in 3% yield. The yield is thereby comparable to that of **2**, indicating that similar processes take place. Hence, the small changes in ligand size seem to have only a minor influence on the reaction cascade, leading to a similar outcome of metastable subhalides (Figure 4).

**Figure 4:**
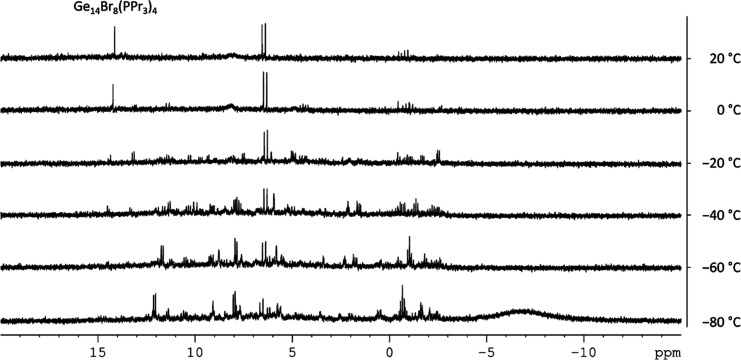
Excerpt of ^31^P-VT-NMR of the metastable solution measures in THF-*d*_8_. The full spectrum is depicted in Figure S7.

However, the small changes in ligand size have a great influence on the solubility; thus, in contrast to **2**, **3** is well soluble in organic solvents like THF and toluene, and thus further investigations are possible. As **3** is a metastable subhalide on the way to elemental germanium, it exhibits a high germanium content of 45 wt %, and only in nonmetal ligands, we reasoned that it should be suitable as a precursor for laser-induced writing of elemental germanium. Therefore, we applied a similar setup as it was used for gold printing starting from Au_32_(P^*n*^Bu_3_)_12_Cl_8_.^18^

Since **3** is unstable in air, the printing process requires inert conditions. This was achieved by storing the ink solution in a sealed vial attached to the glass substrate (Figure S10a). Patterning is performed in a microscopic laser scanning setup, where a 488 nm laser is focused onto the interface between the substrate and ink. Controlled structures of granulated Ge are written on the substrate using a home-built laser scanning system with a 100 × 100 μm^2^ piezo stage, as described in detail in previous work.^18^ Some simple shapes, such as lines or rectangles, are depicted in Figure 5a. Through focused illumination, we achieve line widths down to approximately 2 μm, approaching the optical diffraction limit of the setup around 500 nm (spot diameter *d* = 1.22 × λ/NA ≈ 0.9 μm), and we have achieved a line width of 500 nm and below for gold-printed structures. However, this was only reached by a power and dwell time adjustment together with a dilution series of the dried ink, which was stable in air. Using a home-built scanning system with a commercial controller (HydraLab X1), arbitrary patterns can be realized, such as the representation of the Ge_14_Br_8_(P^*n*^Pr_3_)_4_ cluster structure depicted in Figure 5b. Owing to the air sensitivity of **3**, a residue forms during postprocessing, presumably consisting of GeO_2_, which can be dissolved by brief etching in aqueous pH = 10 KOH solution (cf. Figures S10b,c, S11, and S12). In principle, Ge surface oxidation and residue formation could be avoided under fully inert conditions, which are outside of our experimental capabilities but feasible in an industrial setting. The printed structure consists of elemental germanium as proven by EDX measurements (Figures S13–S18 and Tables S5 and S6). For electrical characterization, the Ge films are contacted by Ti/Au top contact electrodes by using optical lithography to form conductive channels (Figure S19). From temperature-dependent *I*–*V* characteristics in the linear low-voltage regime (Figure 5c), conductance values *G* are extracted as d*I*/d*V*, from which the conductivity ρ is derived via $\rho =G\times \frac{l}{w\times h}$ , with *l*, *w*, and *h* being the channel length, width, and height, respectively (see Figures S20 and S21 and Table S7). As shown in Figure 5d, the conductivity increases with the temperature, which is typical for semiconducting materials.

**Figure 5:**
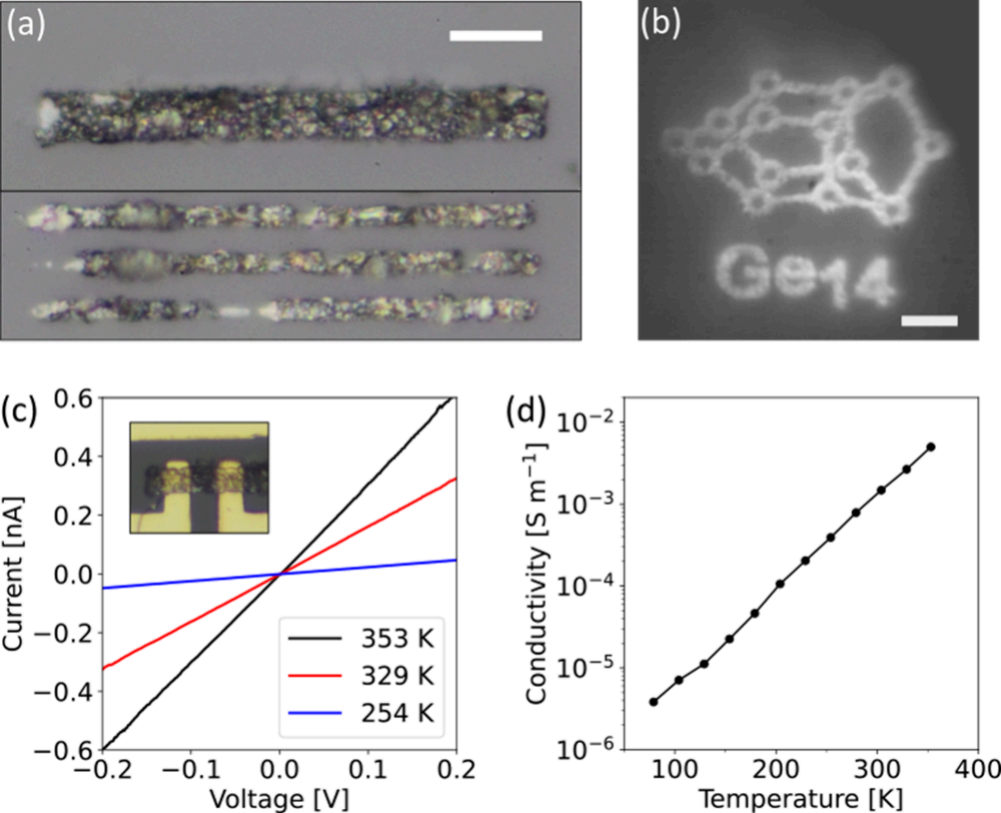
Direct laser writing of elemental Ge microstructures from an ink solution of **3** in THF (10 mg/mL). (a) Rectangular and line prints of granulated Ge after postprocessing in ambient conditions (cf. Figure S11). Scale bar: 10 μm. (b) Laser-written artistic representation of the Ge_14_ cluster core structure. The micrograph was taken directly in the laser writing system and therefore represents the printing quality under inert conditions. Scale bar: 10 μm. (c) *I*–*V* characteristics for three exemplary temperatures used for conductivity extraction. *I*–*V* characteristics of all applied temperatures (Figure S19) as well as nonlinear *I*–*V* characteristics of four channels in the ±5 V regime (Figure S23) are provided in the Supporting Information. (d) Temperature-dependent conductivity measurements in vacuum, demonstrating a positive correlation between temperature and conductivity. An Arrhenius-like representation of the data is provided in the Supporting Information (Figure S22).

The temperature-dependence is in good agreement with previous reports on amorphous Ge (cf. Figure S22).^19^ To the best of our knowledge, this constitutes the first demonstration of Ge patterning by laser direct writing, highlighting a promising application of soluble Ge nanoclusters for additive microfabrication.

## 3 Conclusions

Ge_14_Br_8_(P^*n*^Pr_3_)_4_ is introduced as a new, soluble metalloid germanium cluster obtained by controlled disproportionation of donor-stabilized GeBr, isostructural to Ge_14_Br_8_(PEt_3_)_4_ but with significantly improved solubility due to the tripropylphosphine ligand. Its well-defined cage-like structure, combined with good solubility in organic solvents, enables detailed spectroscopic characterization and reveals an average oxidation state below +1 for germanium, confirming its subvalent, metalloid nature. The improved solubility of Ge_14_Br_8_(P^*n*^Pr_3_)_4_ unlocks its application as a high germanium content precursor for direct laser writing of elemental Ge, yielding micrometer-scale conductive patterns that exhibit temperature-activated electric conductivity. These results demonstrate a route from metalloid Ge clusters to patterned semiconducting structures, highlighting their potential for the additive microfabrication of germanium-based electronics.

## 4 Experimental Section

### 4.1 General Considerations

All reactions were carried out under inert conditions with argon using a standard Schlenk technique. Reagents used for the phosphine synthesis were distilled beforehand. Mg was stored under an argon atmosphere. THF and Et_2_O were dried with sodium, and MeCN with P_4_O_10_ and pentane was dried with CaH_2_. All solvents were distilled prior to use. Germanium pieces (3.2 mm) at 99.999% were bought from Thermo Fisher Scientific. NMR spectroscopic measurements were done at a Bruker AVIIIHD-300, AVII+400, or AVII+500 spectrometer. The chemical shifts are given in ppm against the external standard SiMe_4_. C_6_D_6_ and THF-*d*_8_ were dried with 3 Å molecular sieves.

Crystallographic data were collected on a Bruker APEX II DUO diffractometer equipped with an IμS microfocus sealed tube and QUAZAR optics for monochromated MoKα radiation and equipped with an Oxford Cryosystems cryostat. A semiempirical absorption correction was applied using the program SADABS. The structure was solved by direct methods and refined against F^2^ for all observed reflections. Programs used were SHELXT and SHELXL within the Olex2 program package.^20–22^ The supplementary crystallographic data (CCDC number 2526546) can be obtained online free of charge at www.ccdc.cam.ac.uk/conts/retrieving.html or from Cambridge Crystallographic Data Centre, 12 Union Road, Cambridge CB21EZ; Fax: (+44)­1223–336–033; or deposit@ccdc.cam.ac.uk.

#### 4.1.1 Synthesis of P^*n*^Pr_3_

P^*n*^Pr_3_ was synthesized as described in the literature.^23^

#### 4.1.2 Synthesis of GeBr (P^*n*^Pr_3_/THF)

GeBr was synthesized using a home-built co-condensation apparatus.^11^ Elemental germanium (pur. 99.999%) was placed in a graphite reactor. The pressure in the apparatus was lowered to 5 × 10^–6^ mbar. The reactor is then heated inductively to 1600 °C by using a high-frequency alternating field via an induction coil. As the THF/P^*n*^Pr_3_-mixture (9:1, *V*_total_ = 200 mL) is condensed onto a stainless-steel surface cooled by liquid nitrogen, gaseous HBr was introduced into the reactor, initiating the reaction with the liquid germanium. The flow rate of HBr was 0.2 mmol/min, and the gaseous GeBr is co-condensed with the solvent mixture to give a solid matrix. The side product hydrogen is continuously pumped off via a pumping system. After the consumption of 40 mmol of HBr, the reaction was terminated. After the apparatus was flushed with gaseous nitrogen, the matrix was melted by heating the metal surface to −78 °C. Afterward, the metastable solution was pressed out of the apparatus and stored at −80 °C.

#### 4.1.3 Synthesis of Ge_14_Br_8_(P^*n*^Pr_3_)_4_ (**3**)

A −78 °C cold metastable solution of Ge­(I)Br in THF/P^*n*^Pr_3_ was heated to room temperature and stirred at room temperature for 12 h. On heating, the color of the metastable solution changes from orange red to dark red. Afterward, the solvent is removed to give a dark red oily residue. Excess phosphine was removed by repeated washing with pentane. Extraction with acetonitrile led to the precipitation of a yellow solid. After sedimentation and filtration, a clear, dark red solution is obtained. Further purification was achieved by storing the solution at −30 °C where a red solid precipitates. After filtering and concentrating, dark red crystals of Ge_14_(P^*n*^Pr_3_)_4_Br_8_ (**3**) are obtained after several days by storing the solution at 6 °C (192 mg, 0.0836 mmol, 3%).

^31^P-NMR (300 MHz, THF-*d*_8_, δ, ppm): 14.16 (s, 4P)

## Supplementary Material



## Notes

The following files are available free of charge at https://pubs.acs.org/doi/ and includes analytical data and spectra, crystallographic data and DFT calculations. (PDF). The Supporting Information is available free of charge at https://pubs.acs.org/doi/10.1021/acs.inorgchem.6c00575.

Additional pictures, EDX/SEM, high resolution massspectrometry, UV–vis, NMR, crystallographic section, direct laser writing (DLW) of Ge structures, EDX/SEM of the printed germanium, optical lithography, and electrical characterization (PDF)
