# Supplementary material for: Ge14Br8(P n Pr3)4: A Material for Laser-Induced Printing of Elemental Germanium
Source: Inorg Chem. 2026 May 6;65(19):10616–21. doi: 10.1021/acs.inorgchem.6c00575 (PMC13188054; doi:10.1021/acs.inorgchem.6c00575)
Supplement: Supplementary file 1 [file ic6c00575_si_001.pdf]

Supporting Information

# $Ge_{14}Br_8(P^nPr_3)_4$: A Material for Laser Induced Printing of Elemental Germanium

Enes Ünver[1#], William Roberts[2#], Martin Eberle[2], Kai Braun[2], Marcus Scheele[2], Andreas Schnepf[1,*]

[1]Institute of Inorganic Chemistry, Universität Tübingen, Auf der Morgenstelle 18, D-72076 Tübingen, Germany

[2]Institute of Physical and Theoretical Chemistry, Universität Tübingen, Auf der Morgenstelle 18, D-72076 Tübingen, Germany

[#] These authors contributed equally.

[*] Prof. Dr. A. Schnepf, Tel.: Int. Code +49 (7071) 29 – 76635; Fax: Int. Code +49 (7071) 28 – 2436; Email: andreas.schnepf@uni-tuebingen.de

Table of contents

1. Pictures
2. EDX/SEM
3. High resolution mass spectrometry
4. UV/Vis
5. NMR
6. Crystallographic Section
7. Direct Laser Writing (DLW) of Ge Structures
8. EDX/SEM of the printed germanium
9. Optical Lithography
10. Electrical Characterization

1. Pictures

a) b)

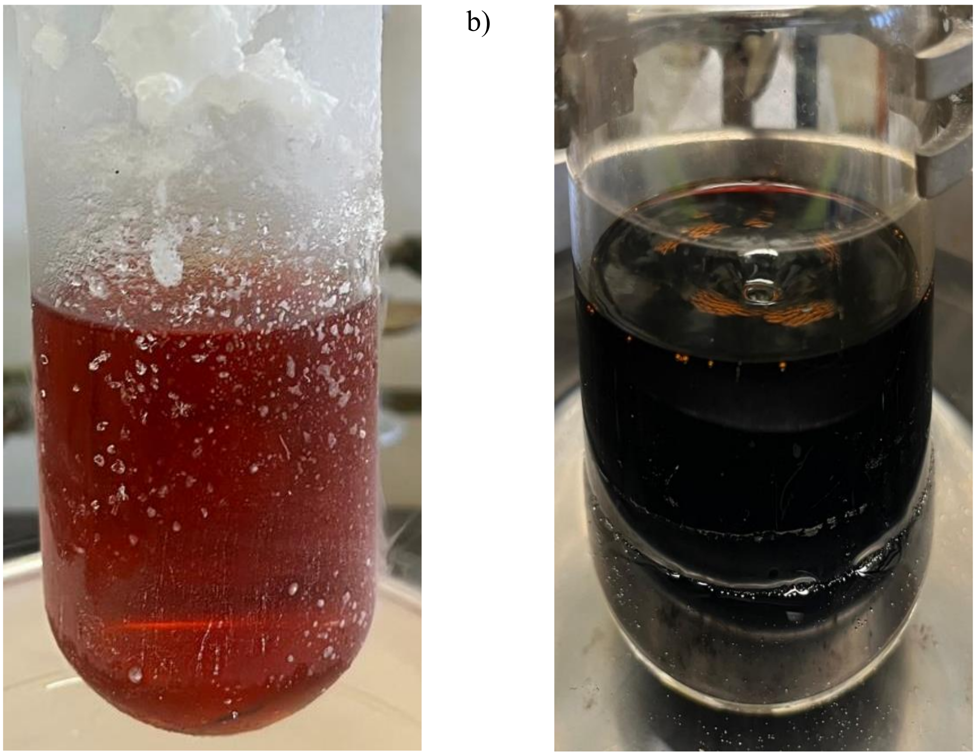

Figure S1: a) Metastable solution at −78 °C. b) Metastable solution at room temperature.

2. EDX/SEM

EDX analysis was performed at a HITACHI SU8030 scanning electron microscope.

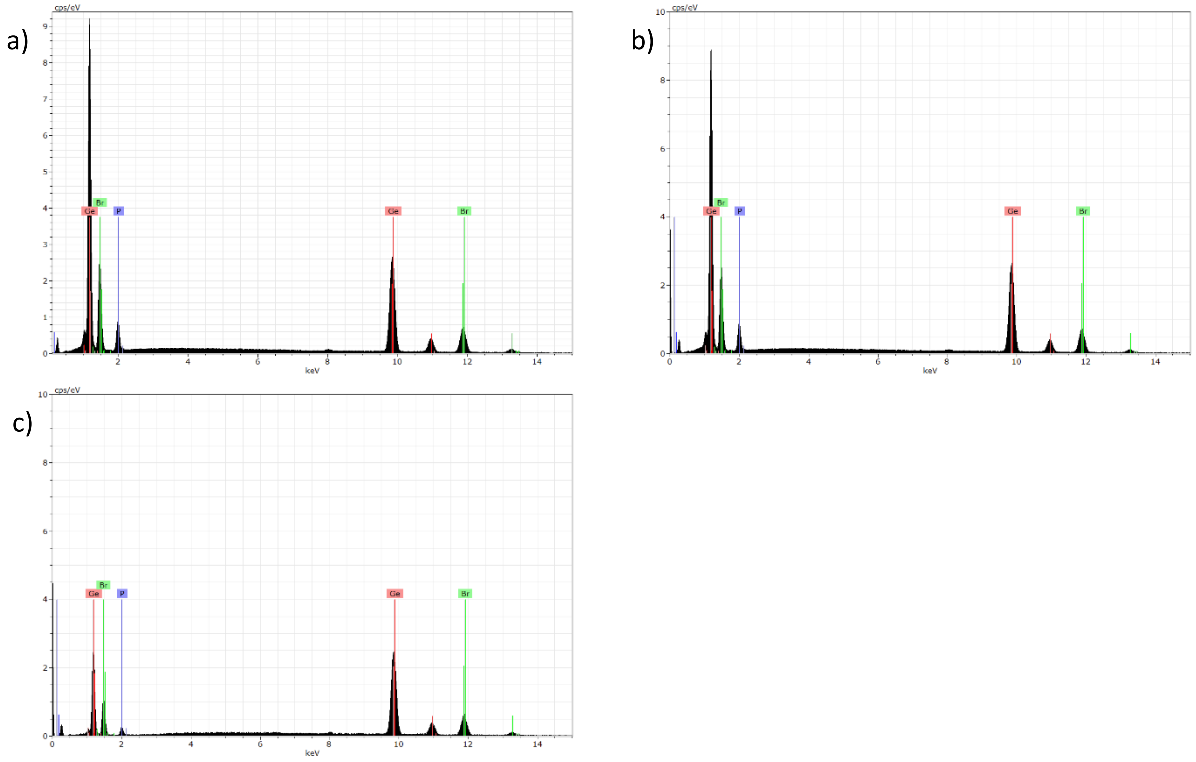


Figure S2: EDX spectra of the points 14 (a) and 16 (b) and 20 (c)

Table S1: EDX results of point 14

| Element | Norm Wt. % | Norm Wt. % calculated | Norm At % | Norm. At % calculated | Error (Wt % 3 σ) |
|---|---|---|---|---|---|
| Germanium | 54,43 | 57,11 | 53,26 | 53,84 | 3,88 |
| Bromine | 33,54 | 35,92 | 31,56 | 30,77 | 2,67 |
| Phosphorus | 6,25 | 6,96 | 15,18 | 15,38 | 0,88 |

Table S2: EDX results of point 16

| Element | Norm Wt. % | Norm Wt. % calculated | Norm At % | Norm. At % calculated | Error (Wt % 3 σ) |
|---|---|---|---|---|---|
| Germanium | 54,02 | 57,11 | 50,36 | 53,84 | 3,60 |
| Bromine | 33,51 | 35,92 | 32,18 | 30,77 | 2,67 |
| Phosphorus | 7,99 | 6,96 | 17,45 | 15,38 | 0,96 |

Table S3: EDX results of point 20

| Element | Norm Wt. % | Norm Wt. % calculated | Norm At % | Norm. At % calculated | Error (Wt % 3 σ) |
|---|---|---|---|---|---|
| Germanium | 49,66 | 57,11 | 55,54 | 53,84 | 3,84 |
| Bromine | 28,97 | 35,92 | 29,44 | 30,77 | 2,51 |
| Phosphorus | 5,73 | 6,96 | 15,02 | 15,38 | 1,03 |

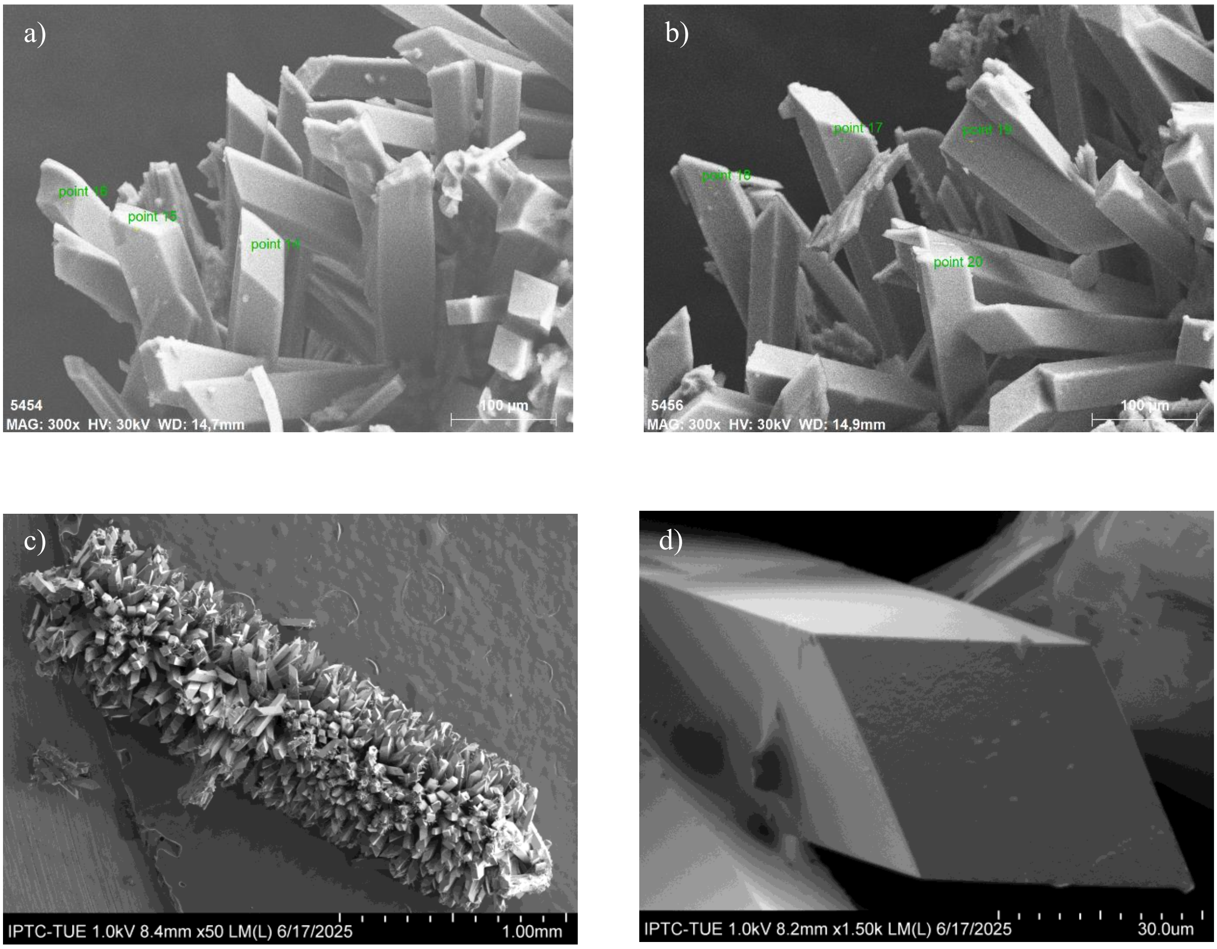


Figure S3: SEM Images of **3**. a + b) SEM images with chosen points of the EDX measurement. c) Crystals accumulation. d) Zoom on one crystal.

3. High resolution mass spectrometry

The HR-ESI-APCI-APPI-MS was measured with a Bruker AmaZon SL with an ion trap analyser. Dry crystals of **3** (approx. 40 µg) were dissolved in THF (1 mL) and directly injected into the device using a syringe pump.

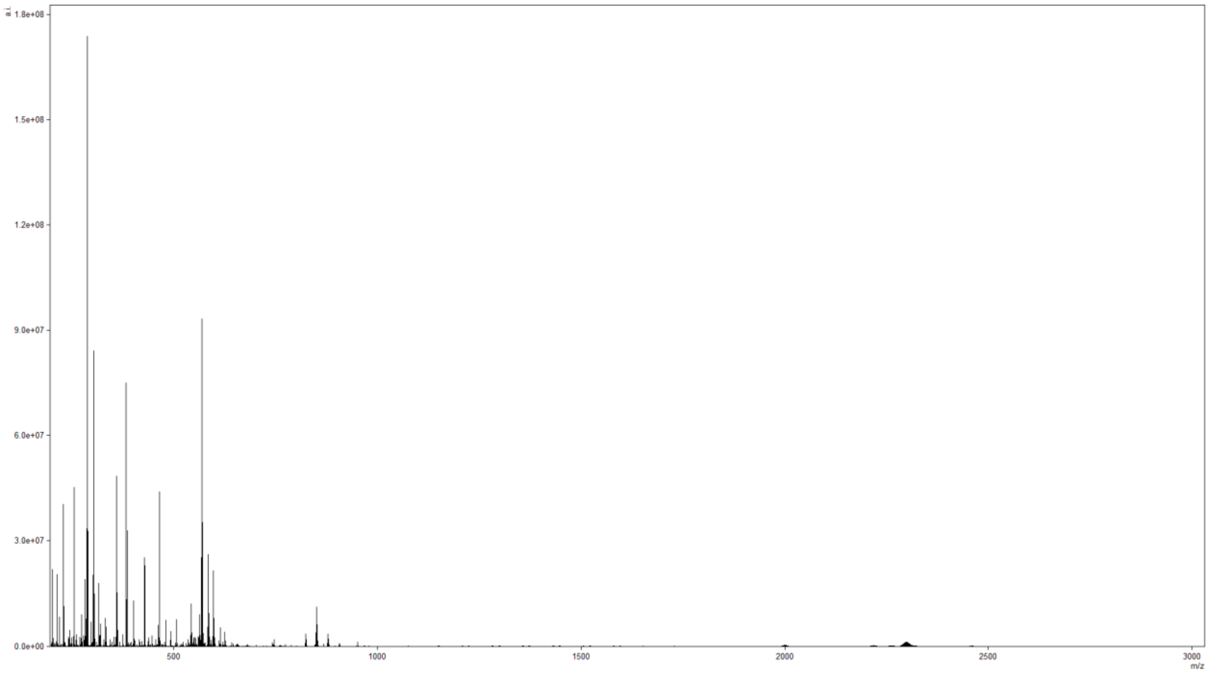


Figure S4: Overview of the HR-ESI-APCI-APPI-MS of **3**

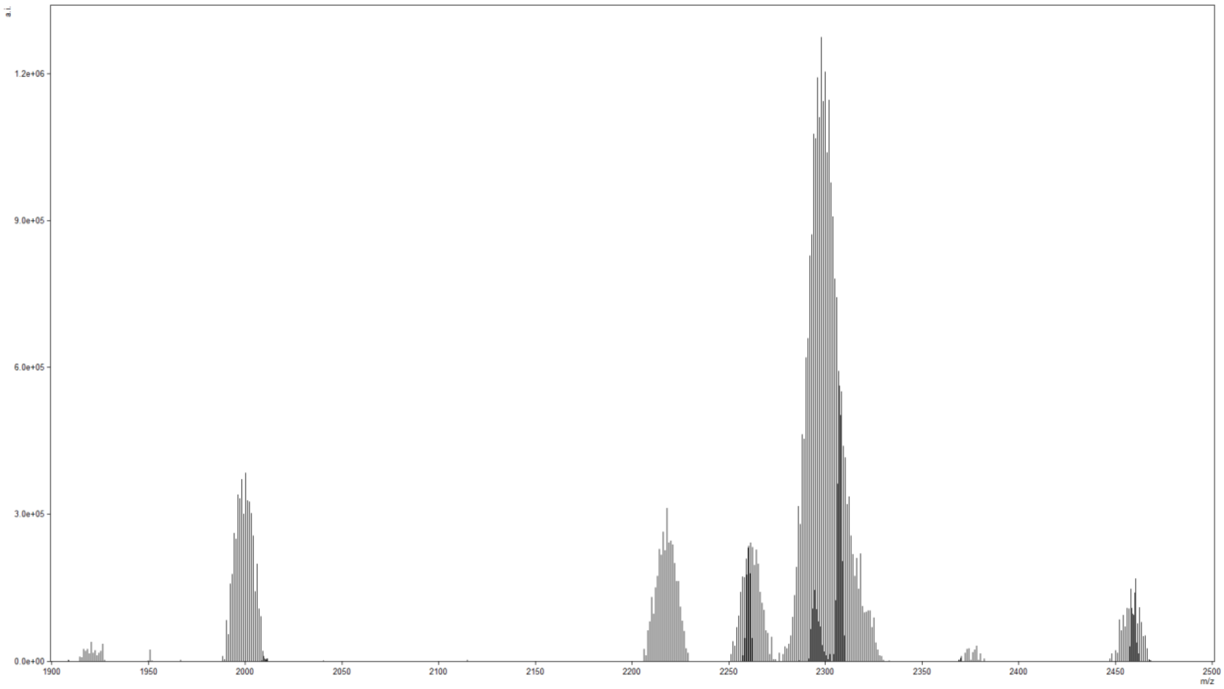


Figure S5: Excerpt of the HR-ESI-APCI-APPI-MS of **3**

4. UV/Vis

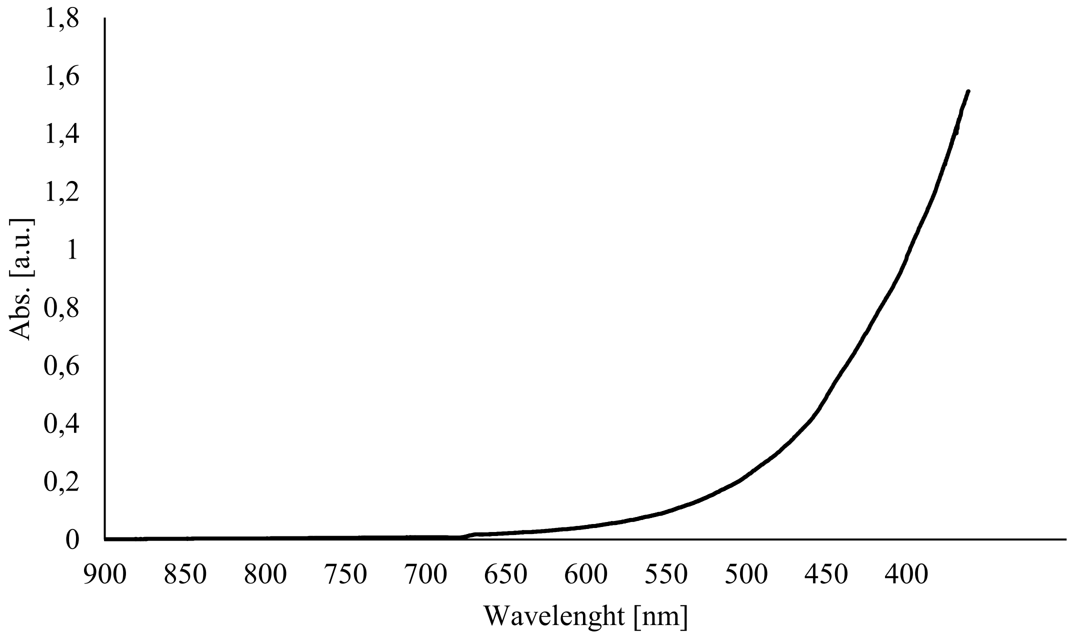


Figure S6: UV/Vis spectra of **3** measured in THF.

5. NMR

NMR spectroscopic measurements were done with a Bruker AVIIIHD-300 spectrometer. The chemical shifts are given in ppm against the external standards SiMe4 (1H, 13C) and H3PO4 (85%, 31P). THF-d8 were dried with NaK alloy.

Variable temperature NMR spectroscopic measurements were performed on a Bruker AVII+500 spectrometer. Therefore, 0.3 mL of the −78 °C cold metastable solution was transferred in a syringe into a pre-cooled NMR tube with a THFd8 lock capillary. The content of the NMR tube was then melted under reduced pressure and heated in 10 °C increments in the spectrometer.

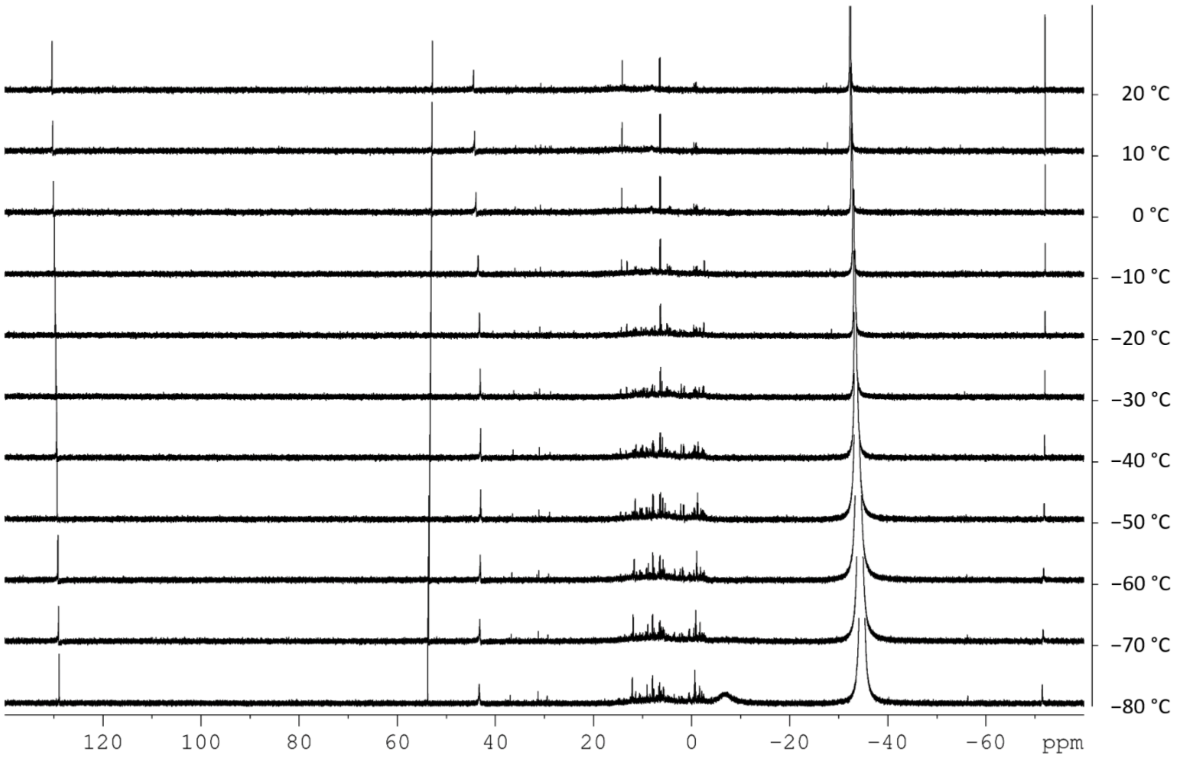


Figure S7: VT-$^{31}$P-NMR-Spectrum of the metastable solution (GeBr/THF/P$^{n}$Pr$_3$) starting from −80 °C bottom to 20 °C top in 10 °C increments.

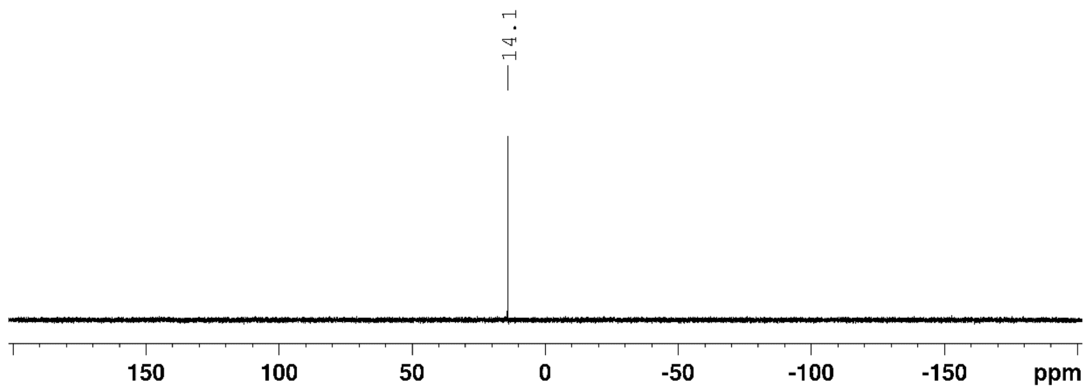


Figure S8: 31P-NMR of **3** measured in THF-d8 at room temperature with the cluster signal at 14.1 ppm.

6. Crystallographic Section

Table S4 Crystal data and structure refinement for **3**.

| | |
|---|---|
| Identification code | 9 |
| Empirical formula | $C_{38}H_{87}Br_8Ge_{14}NP_4$ |
| Formula weight | 2337.50 |
| Temperature/K | 99.77 |
| Crystal system | orthorhombic |
| Space group | $P2_12_12_1$ |
| a/Å | 15.4766(9) |
| b/Å | 16.6657(10) |
| c/Å | 28.2944(16) |
| α/° | 90 |
| β/° | 90 |
| γ/° | 90 |
| Volume/Å$^3$ | 7297.9(7) |
| Z | 4 |
| ρcalcg/cm$^3$ | 2.127 |
| μ/mm$^{-1}$ | 10.166 |
| F(000) | 4440.0 |
| Crystal size/mm$^3$ | 0.079 × 0.077 × 0.061 |
| Radiation | MoKα (λ = 0.71073) |
| 2Θ range for data collection/° | 4.604 to 56.598 |
| Index ranges | $-20 \leq h \leq 20$, $-22 \leq k \leq 22$, $-36 \leq l \leq 37$ |
| Reflections collected | 143690 |
| Independent reflections | 18121 [$R_{int}$ = 0.0560, $R_{sigma}$ = 0.0360] |
| Data/restraints/parameters | 18121/0/599 |
| Goodness-of-fit on F$^2$ | 1.040 |
| Final R indexes [I>=2σ (I)] | $R_1$ = 0.0303, $wR_2$ = 0.0690 |
| Final R indexes [all data] | $R_1$ = 0.0372, $wR_2$ = 0.0714 |
| Largest diff. peak/hole / e Å$^{-3}$ | 1.44/−0.60 |
| Flack parameter | 0.013(4) |

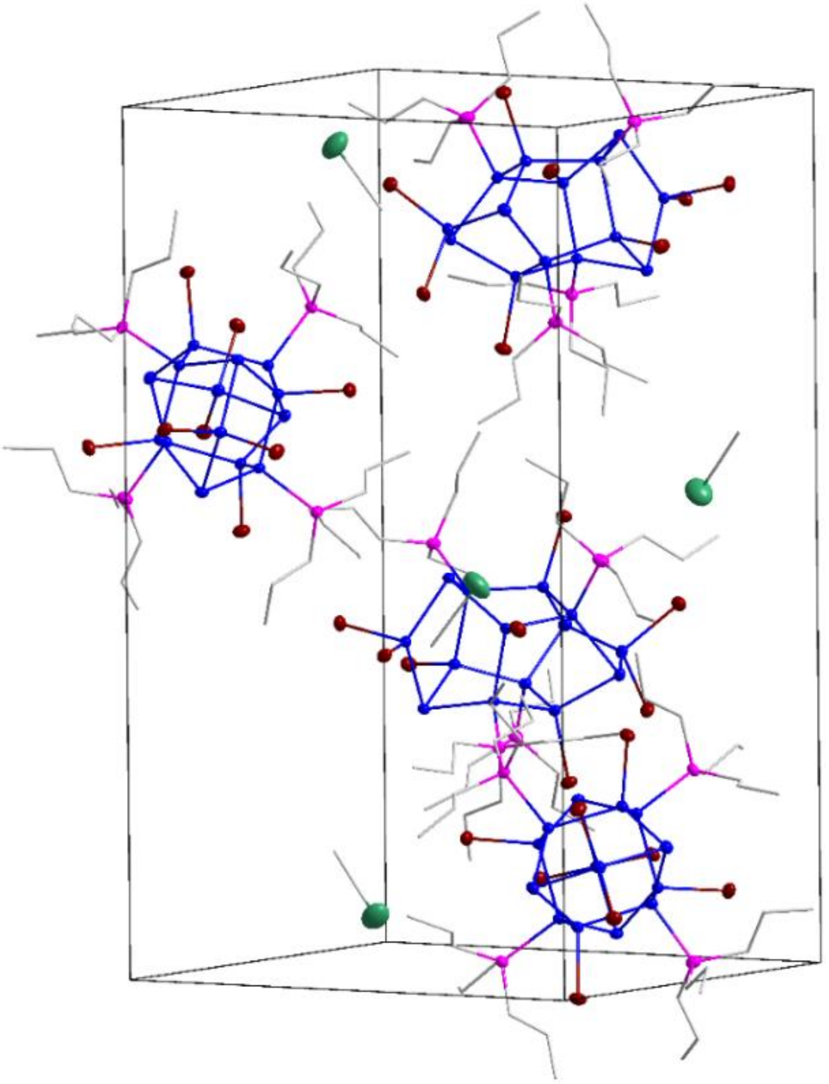

Figure S9: Arrangement of **3** and MeCN in the crystal.

7. Direct Laser Writing (DLW) of Ge Structures

DLW of Ge structures was performed in a homebuilt confocal microscopy setup. To ensure quasi-inert conditions, an Eppendorf vial was cut and mounted on a coverslip (Menzel Gläser, 22×22 mm$^2$) using an optical adhesive. The vial was filled with ink and sealed in a glovebox environment. A 488 nm CW laser ($P_{opt}$ = 1.7 mW) was focused onto the sample through a NA=0.7 objective. Patterning was performed using a homebuilt programmable system (HydraLitho). The 100×100 µm$^2$ scanning range was divided into 256×256 pixels. Exposed pixels were illuminated for 3 ms, with three iterations per print. For details on the instrumentation, the reader is referred to a previous work.[18] After DLW, the sample was processed in ambient conditions. For this, the ink was removed, and the sample immersed in acetone for 1 h to remove the attached vial. Afterwards, the substrate was rinsed with 2-propanol and dried in an air stream. Height profiles were recorded with a Bruker DektakXT profilometer.

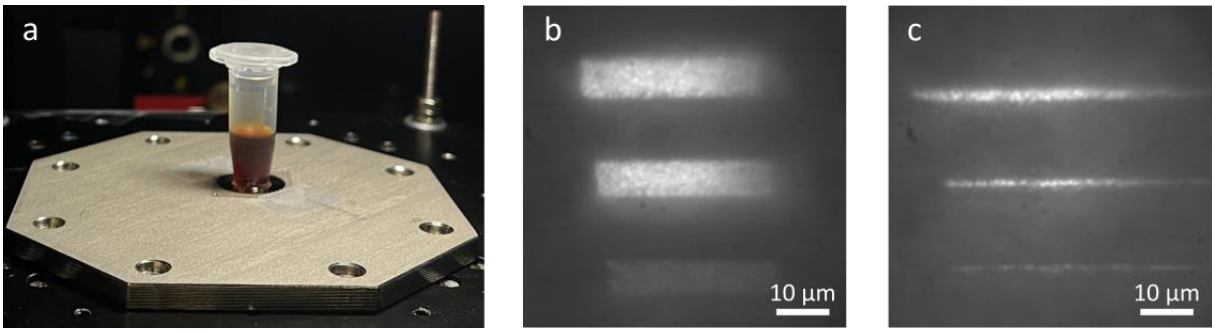


Figure S10: DLW of Ge structures from a solution of **3** in THF (10 mg/mL). a) Sample configuration for DLW in quasi-inert conditions. b) Rectangular prints, recorded after printing under quasi-inert conditions. c) Line prints, recorded after printing under quasi-inert conditions.

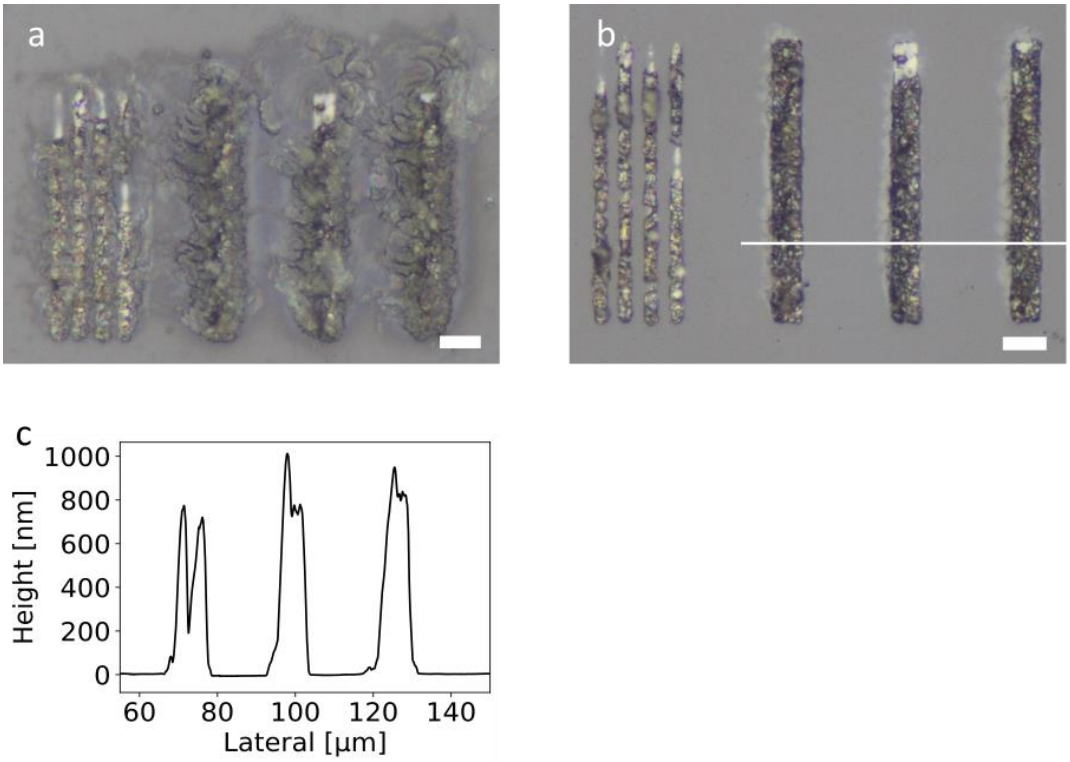


Figure S11: Top view of laser-written granulated Ge structures from THF-based ink as depicted in Fig. S10b-c after post-processing under ambient conditions. a) Top view of the printed Ge structures, covered with a residue. Scale bar: 10 µm. b) Etching in aqueous KOH (pH=10) for 15 s effectively dissolves the residue. Scale bar: 10 µm. c) Height profile marked in b).

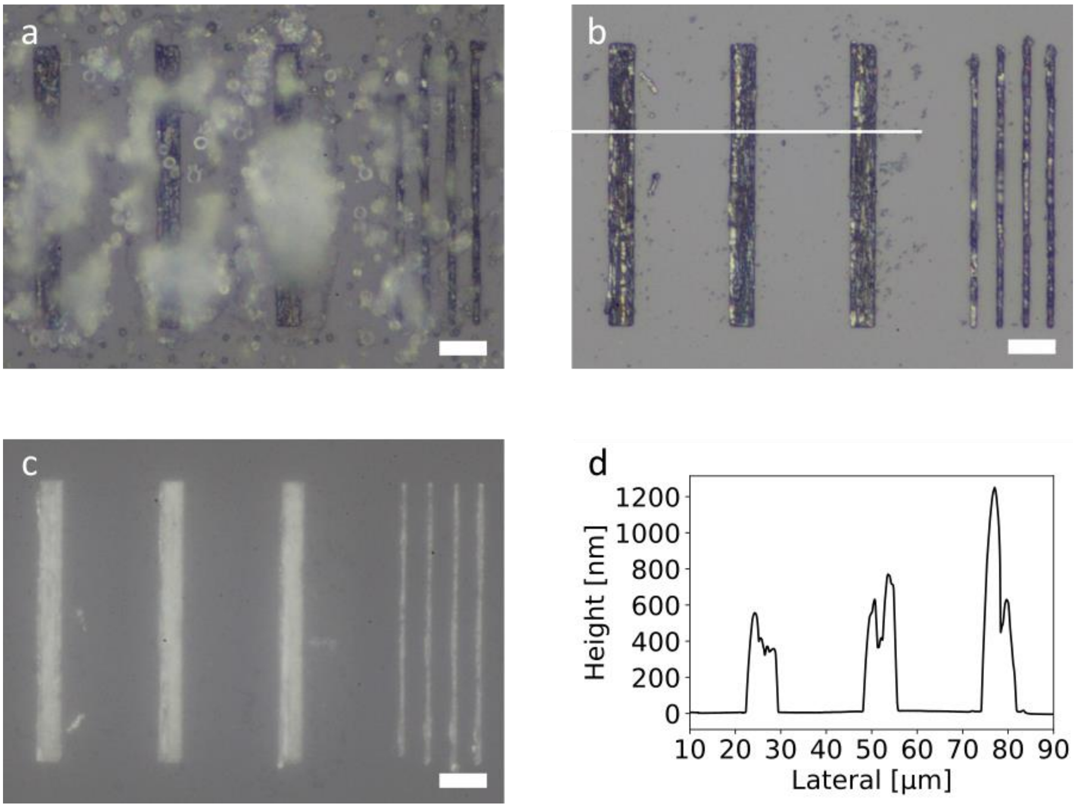


Figure S12: Ge structures written from a MeCN extract of the metastable solution as ink after post-processing under ambient conditions. In principle, this approach yields superior printing quality, suggesting potential for solvent optimization. However, the metastability of the MeCN extract may impose challenges regarding ink stability. a) Top view of the printed Ge structures, covered with residue. Scale bar: 10 µm. b) Etching in aqueous KOH (pH=10) for 15 s effectively dissolves the residue. Scale bar: 10 µm. c) Bottom view of image b). Scale bar: 10 µm. d) Height profile marked in b).

8. EDX/SEM of the printed germanium

The printed germanium was examined via EDX spectroscopy and SEM. Therefore, the printed Germanium lines (Figure S12) were contacted with 5 nm Ti layer and 100 nm Au layer electrode patterns via optical lithography and wire bonded with aluminium wire to the sample holder to mitigate charging effects.

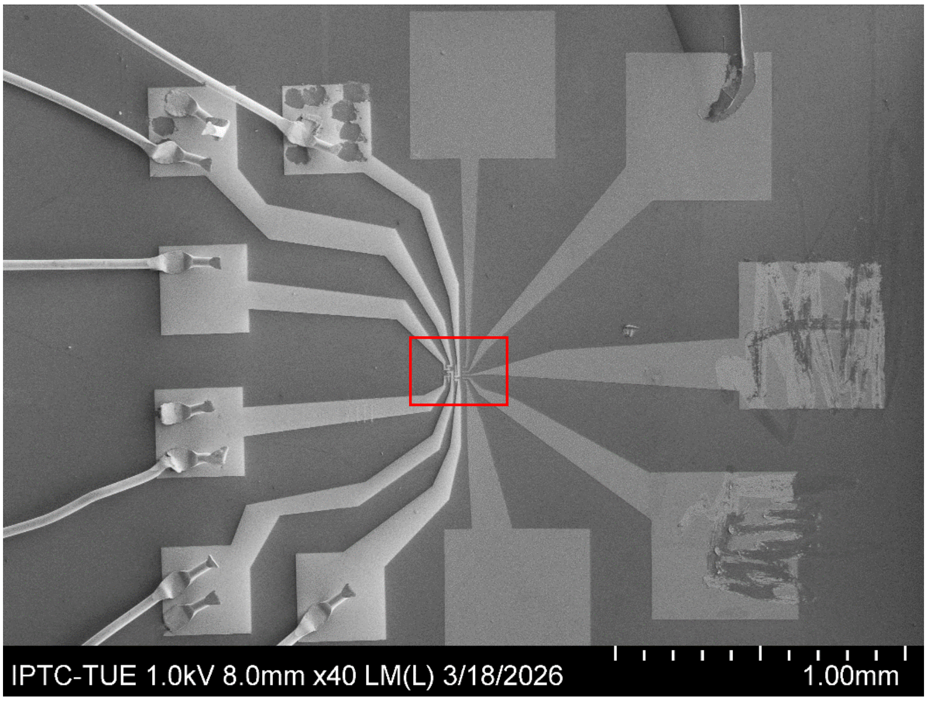


Figure S13: SEM image showing an overview of the measurement object for EDX characterization.

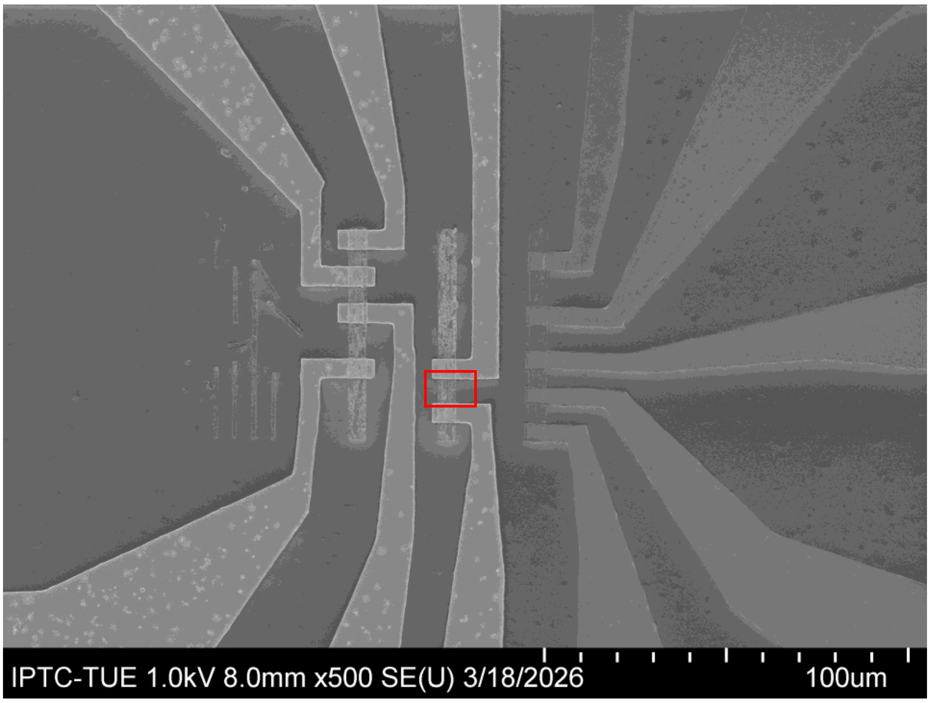


Figure S14: Magnified section of figure S13 as indicated by the red rectangle, showing the contacted germanium prints.

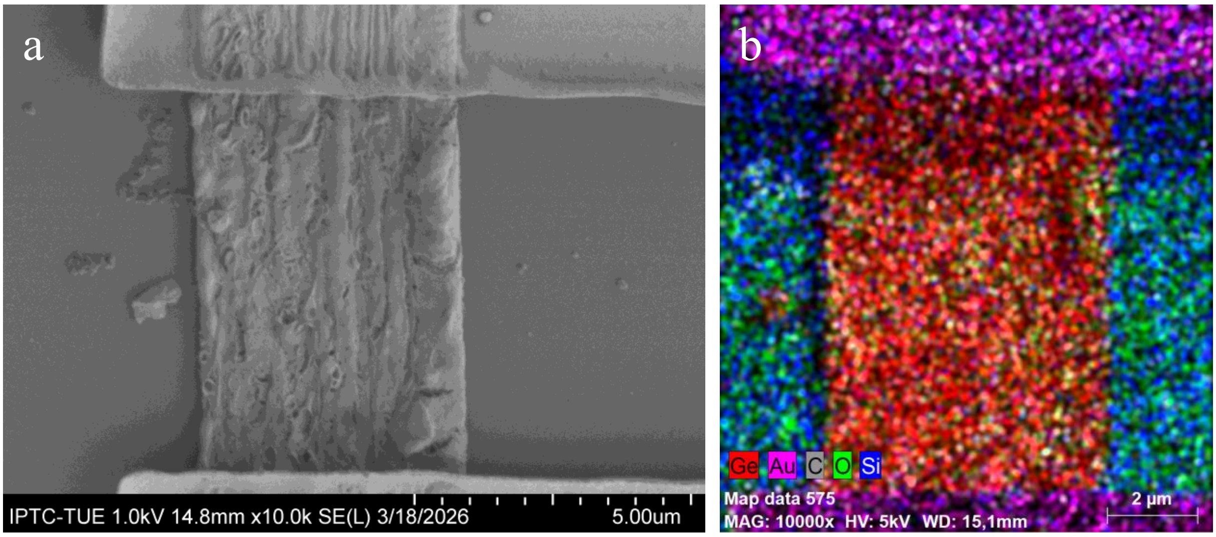

Figure S15: a) Magnified section of figure S14 as indicated by the red rectangle of the gold contacted germanium line. B) EDX map of the area with Ge in red, Au in pink, C in grey, O in green and Si in blue.

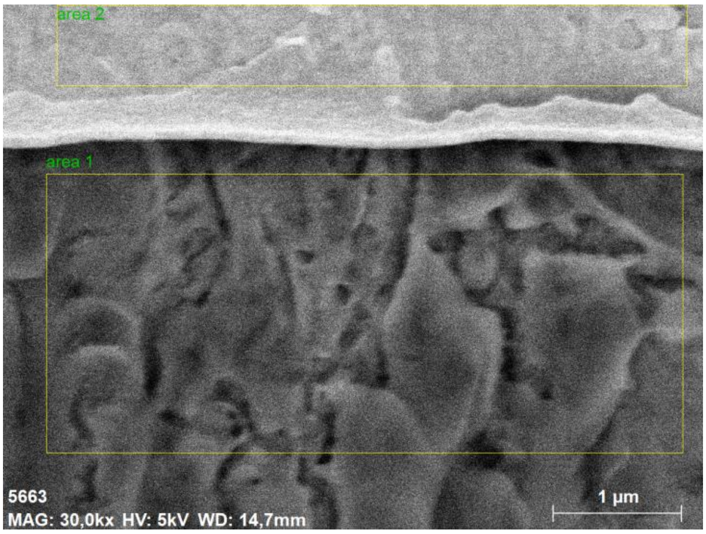

Figure S16: a) Zoom of the SEM image of the gold contacted germanium line with measured area 1 (germanium structure) and area 2 (gold contact). b) EDX of the area 1. We attribute the oxygen contribution to a native oxide layer c) EDX of the area 2, dominated by the overlaying Ti/Au electrode.

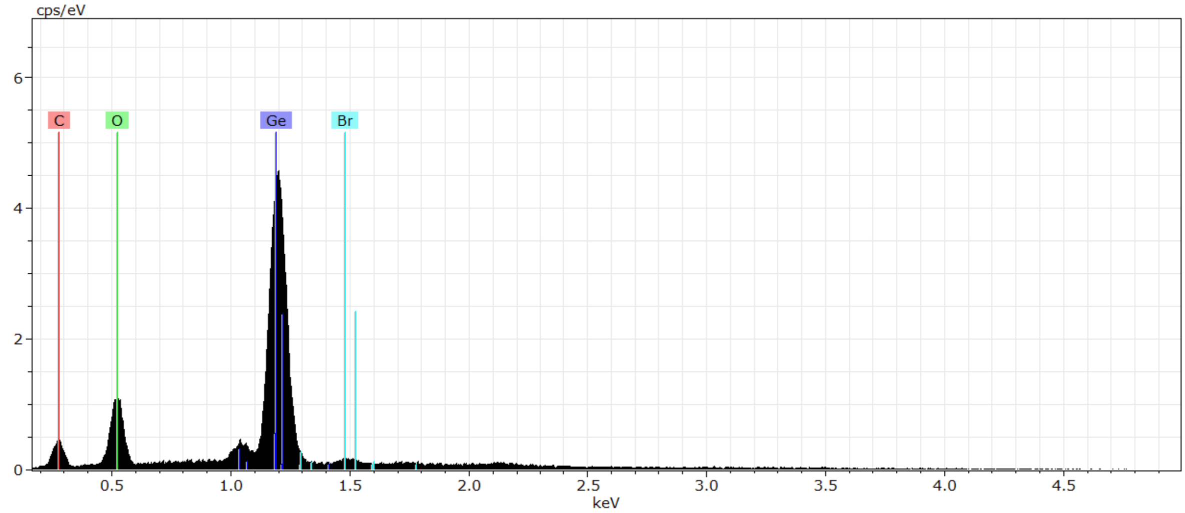


Figure S17: EDX of the area 1. We attribute the oxygen contribution to a native oxide layer

Table S5: EDX results of Area 1

| Element | Wt. conc. (W. %) | Norm conc. (Wt. %) | Atom conc. (%) | Error 3 σ (Wt %) |
|---|---|---|---|---|
| Germanium | 75,57 | 79,63 | 45,36 | 12,07 |
| Bromine | 1,63 | 1,72 | 0,89 | 0,42 |
| Oxygen | 11,59 | 12,21 | 31,57 | 5,15 |
| Carbon | 6,11 | 6,44 | 22,18 | 3,35 |

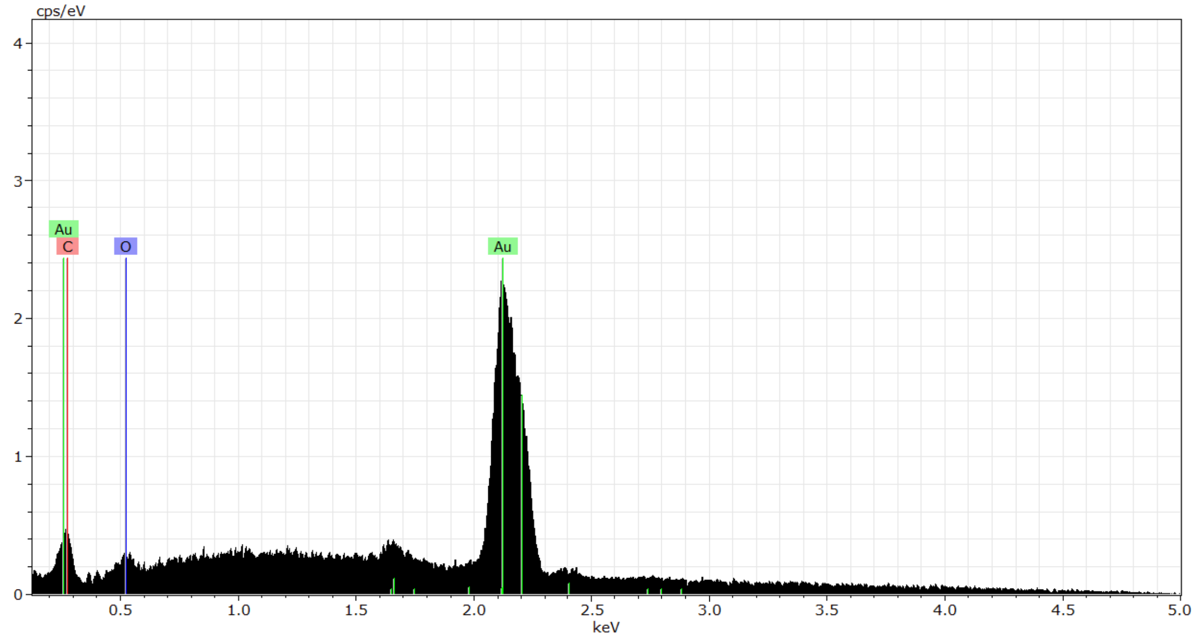


Figure S18: EDX of the area 2, dominated by the overlaying Ti/Au electrode.

Table S6: EDX results of Area 2

| Element | Wt. conc. (W. %) | Norm conc. (Wt. %) | Atom conc. (%) | Error 3 σ (Wt %) |
|---|---|---|---|---|
| Gold | 104,15 | 98,57 | 81,95 | 14,92 |
| Oxygen | 0,45 | 0,43 | 4,35 | 0,42 |
| Carbon | 1,06 | 1,00 | 13,70 | 0,72 |

9. Optical Lithography

Positive photoresist (ma-P 1215, Microresist Technology) was spin-coated at 3000 rpm for 30 s and prebaked on a hot plate at 100 °C for 1 min. The electrode pattern was defined by aligning the sample with a photomask and exposure to 365 nm UV-light using a maskless aligner (Heidelberg Instruments). Development was performed in ma-D 331/S developer (Microresist Technology) for 32 seconds. Metal contacts were formed by electron beam evaporation of a 5 nm adhesive Ti layer and a 100 nm Au layer. The lift-off process was performed by immersing the samples in acetone for 2 h, after which the substrates were rinsed with isopropanol and dried in a nitrogen stream.

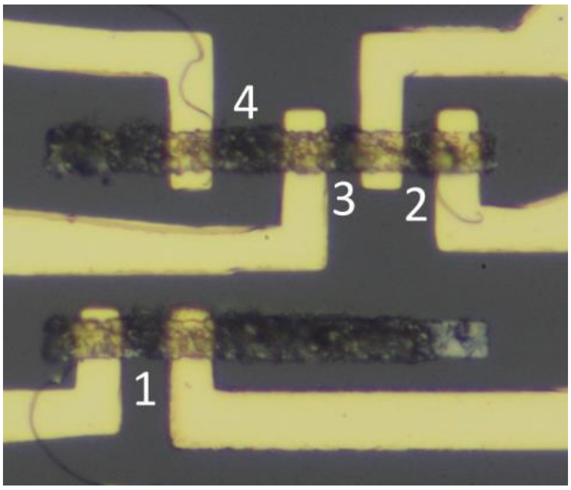


Figure S19: Top Ti/Au contacts applied to the DLW-written Ge structures depicted in Fig. S11b form four conductive channels which are used for electrical characterization. For channel dimensions, see Table S5.

*10.* Electrical Characterization

Electrical measurements were conducted in a Lake Shore Cryotronics CRX-6.5K probe station with a temperature controller model 336, where the samples were contacted by gold-coated tungsten tips connected to a Keithley 2636B source meter.

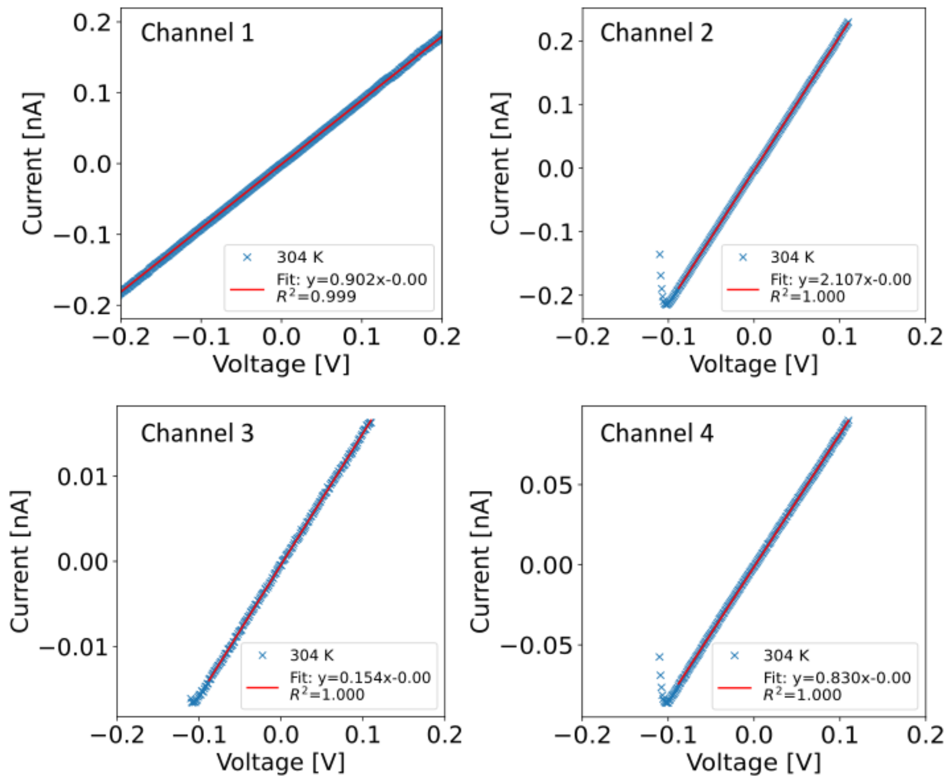


Figure S20: *V*-*I*-characteristics of four Ge channels depicted in Fig. S13, recorded at 304 K in vacuum (<$10^{-4}$ mbar). The conductance values are determined by linear regression and summarized in Table S7.

Table S7: Summarized dimensions, conductance and conductivity at 304 K of the four Ge channels depicted in Fig. S19. Since the height profiles exhibit significant roughness, an approximated height of 700 nm was assumed for all Ge channels, introducing some uncertainty into the resulting conductivity values.

| Channel | Length [µm] | Width [µm] | Height [nm] | Conductance [A/V] | Conductivity [S/m] |
|---|---|---|---|---|---|
| 1 | 6.1 | 5.3 | 700 | $9.0\times10^{-10}$ | $1.3\times10^{-3}$ |
| 2 | 4.2 | 5.3 | 700 | $2.1\times10^{-9}$ | $2.4\times10^{-3}$ |
| 3 | 4.2 | 5.3 | 700 | $1.5\times10^{-10}$ | $1.7\times10^{-4}$ |
| 4 | 9.1 | 5.4 | 700 | $8.3\times10^{-10}$ | $2.0\times10^{-3}$ |

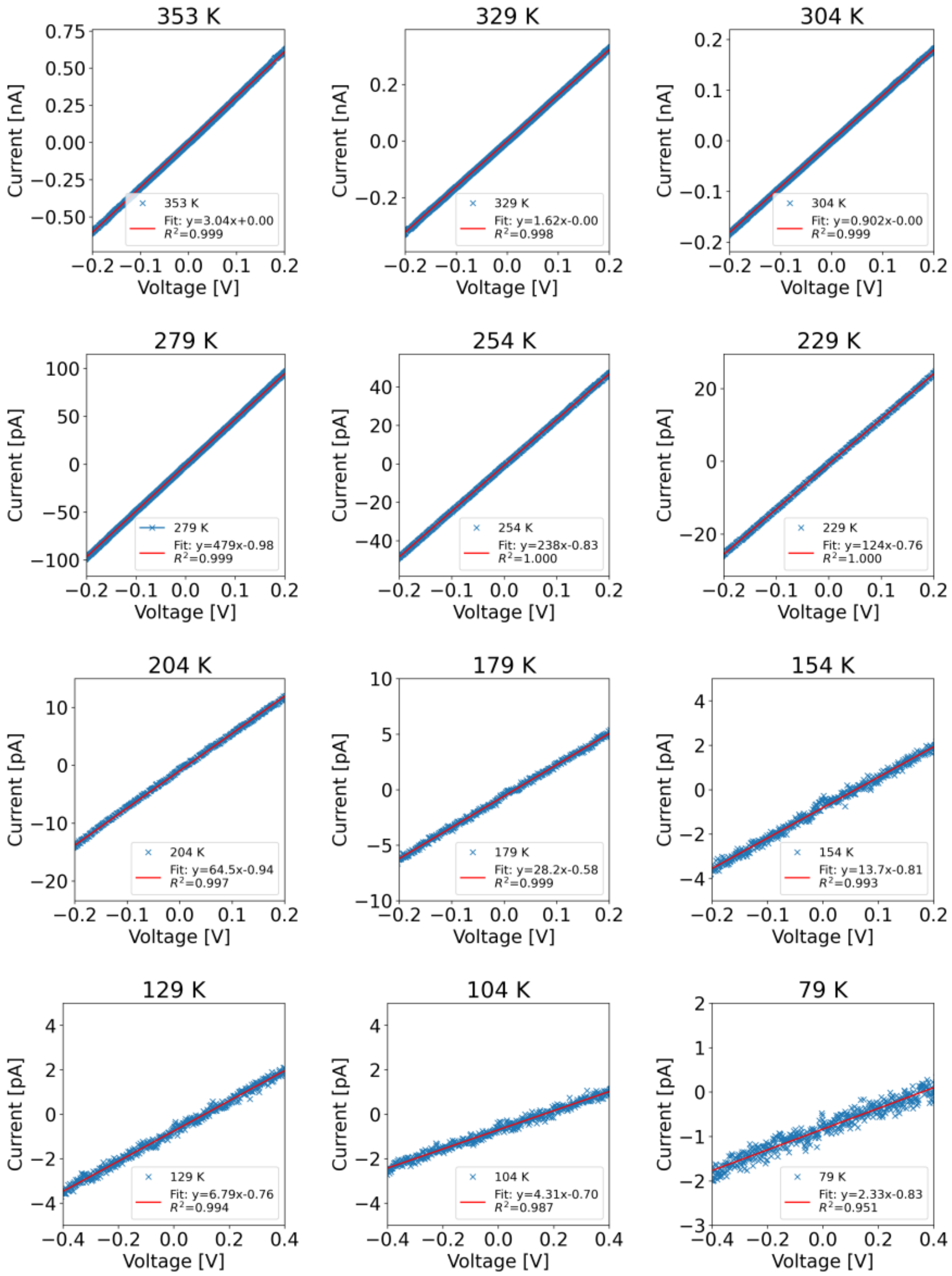


Figure S21: *V*-*I*-characteristics of Ge channel 1 (see Fig. S13) at several different temperatures in vacuum, used for temperature-dependent conductivity investigations (Fig. 5c, Fig. S20).

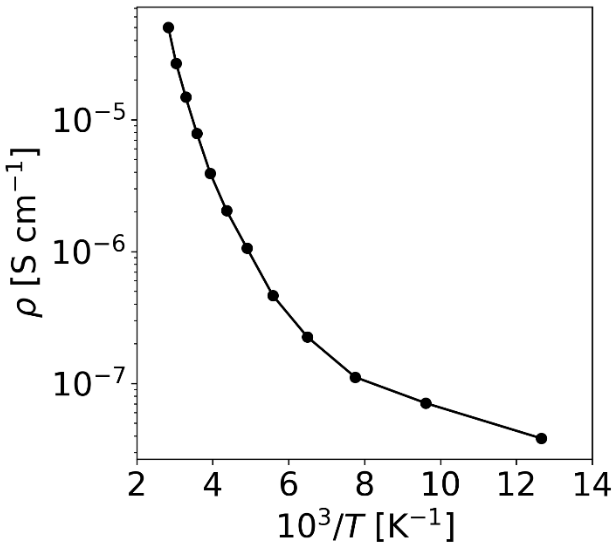


Figure S22: Arrhenius-like representation of the temperature-dependent conductivity values depicted in Fig. 5c. The resulting trend is in good agreement with previous literature reports on amorphous Ge.[19]

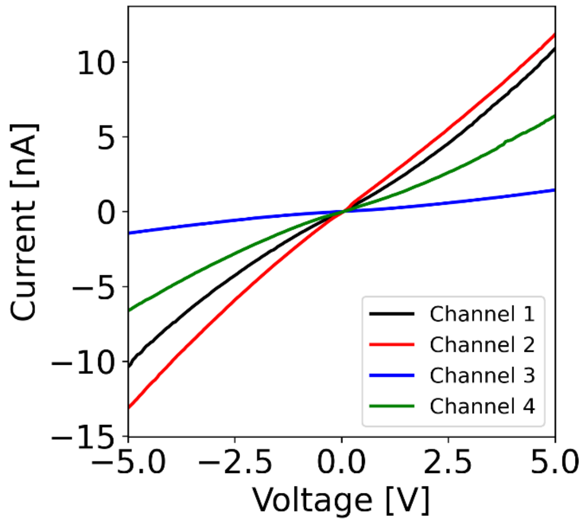


Figure S23: *V*-*I*-characteristics of four Ge channels depicted in Fig. S19, recorded at 304 K in vacuum (<$10^{-4}$ mbar), featuring source voltages of ±5 V. With increasing fields, non-linear *V*-*I*-characteristics are observed.
